# The Peptide-Drug Conjugate TH1902: A New Sortilin Receptor-Mediated Cancer Therapeutic against Ovarian and Endometrial Cancers

**DOI:** 10.3390/cancers14081877

**Published:** 2022-04-08

**Authors:** Jean-Christophe Currie, Michel Demeule, Cyndia Charfi, Alain Zgheib, Alain Larocque, Bogdan Alexandru Danalache, Amira Ouanouki, Richard Béliveau, Christian Marsolais, Borhane Annabi

**Affiliations:** 1 Theratechnologies Inc., 2015 Peel Street, 11th Floor, Montréal, QC H3A 1T8, Canada; jcurrie@theratech.com (J.-C.C.); mdemeule@theratech.com (M.D.); ccharfi@theratech.com (C.C.); alarocque@theratech.com (A.L.); cmarsolais@theratech.com (C.M.); 2 Laboratoire d’Oncologie Moléculaire, Département de Chimie, Université du Québec à Montréal, C.P. 8888, Succ. Centre-Ville, Montréal, QC H3C 3P8, Canada; zgheib.alain@uqam.ca (A.Z.); danalache.bogdan_alexandru@uqam.ca (B.A.D.); ouanouki.amira@uqam.ca (A.O.); beliveau.richard@uqam.ca (R.B.)

**Keywords:** gynecological cancers, peptide-drug conjugates, docetaxel, sortilin

## Abstract

**Simple Summary:**

Tumor heterogeneity, drug resistance, and systemic toxicity are major concerns throughout treatments of gynecological cancers. Off-target toxicity is also a significant issue with traditional cancer medicines, preventing patients’ cancers from being adequately treated with chemotherapeutics alone. As a result, receptor-mediated targeting mechanisms are frequently exploited in conjunction with other drugs and have been the most specific among the group of techniques that have emerged in recent decades. Sortilin (SORT1) is a multifunctional protein whose expression levels have been documented in multiple malignancies, such as ovarian and endometrial tumors. In this study, the anticancer efficacy of TH1902, a peptide-docetaxel conjugate that is internalized in cancer cells through SORT1, was documented on the growth of human ovarian and endometrial cancer cell lines in vitro, as well as in mice’s tumor xenografts models, alone or in combination with carboplatin. This new anticancer drug-conjugate vectorization strategy significantly increases the efficacy of anticancer drugs towards SORT1-positive tumors, leading to a reduction in unwanted side effects. Importantly, TH1902 as a single agent or in combination with carboplatin demonstrates better efficacy than taxanes or carboplatin-taxane-based therapies for the treatment of both ovarian and endometrial cancers.

**Abstract:**

Sortilin (SORT1) receptor-mediated endocytosis functions were exploited for this new approach for effective and safe treatments of gynecological cancers. Here, high expression of SORT1 was found in >75% of the clinically annotated ovarian and endometrial tumors analyzed by immunohistochemistry. Therefore, the anticancer properties of the peptide-drug conjugate TH1902, a peptide that targets SORT1 and which is linked to docetaxel molecules, were investigated both in vitro using ovarian and endometrial cancer cell cultures and in vivo using xenograft models. In vitro, TH1902 inhibited cell proliferation and triggered higher SORT1-dependent cell apoptosis than unconjugated docetaxel did in ES-2 and SKOV3 ovarian cancer cell lines. The uptake of the Alexa^488^-TH19P01 peptide from TH1902 was reduced upon siRNA-mediated silencing of SORT1. In vivo, weekly administration of TH1902 showed better tolerability compared to equivalent docetaxel doses and inhibited tumor growth in ovarian and endometrial xenograft mice models. TH1902 as a single agent inhibited ovarian tumor growth more than either of the unconjugated taxanes or carboplatin. Furthermore, TH1902 combination with carboplatin also demonstrated better efficacy when compared to both taxanes-carboplatin combinations. Overall, TH1902 shows better in vivo efficacy, compared to that of docetaxel and even paclitaxel, against SORT1-positive ovarian and endometrial cancers and could be safely combined with carboplatin.

## 1. Introduction

Epithelial ovarian cancer (EOC) is the most lethal gynecological cancer and represents the seventh most diagnosed cancer among women in the world [1]. Annually, worldwide, 230,000 women will be diagnosed with EOC [2]. Furthermore, the survival 5 years after diagnosis of about 46% is often attributed to the advanced stage at time of diagnosis [3]. EOC is a heterogeneous disease, which consists of at least five types: high-grade serous carcinoma (HGSC), low-grade serous carcinoma (LGSC), mucinous carcinoma (MC), endometrioid carcinoma (EC), and clear-cell carcinoma (CCC). HGSC is the predominant subtype and accounts for 70–80% of the cases, whereas LGSC represents 5% of the cases, MC 3%, EC 10%, and CCC about 10% [4].

Debulking surgery followed by chemotherapy is the standard of care for HGSC. Unfortunately, disease relapse is observed within the first 5 years, with an overall survival of about 30%, for advanced-stage disease [5,6]. Three or more cycles of neoadjuvant chemotherapy (NACT) prior to debulking surgery and adjuvant chemotherapy offer the opportunity to test upfront chemosensitivity and to identify patients at higher risk of relapse, although a lack of consensus remains on who the best candidates for this strategy are [7]. Current chemotherapy regimens used in HGSC are based on a combination of classical chemotherapeutic drugs, namely carboplatin and taxanes (paclitaxel/docetaxel), as well as poly (ADP-ribose) polymerase (PARP) inhibitors, which have also entered into standard treatment for EOC [8].

In contrast to most other cancers in the United States, the incidence and mortality associated with endometrial cancer are increasing [9]. The prognosis is relatively good, with a 5-year survival rate of 80% since many cases are diagnosed at early stages. However, a dismal prognosis is observed in advanced and recurrent endometrial cancer in a significant proportion of patients. Endometrial carcinoma arises from the lining of the uterus and can broadly be divided into two types: endometrioid (Type 1), affecting approximately 80% of patients, and non-endometrioid (Type 2), approximately 20% of patients. The latter includes endometrial serous carcinoma, CCC, and EC [10]. In the past several years, surgical treatment of endometrial cancer has been refined and now incorporates sentinel lymph node mapping, along with the standard, minimally invasive removal of the uterus, fallopian tubes, and ovaries [11]. Adjuvant chemotherapy, with or without radiation, can be used to treat patients with high-risk endometrial cancers undergoing surgery [12]. Recently, overexpression/gene amplification of human epidermal growth factor receptor 2 (HER2) in 20–40% of patients with type 2 endometrial cancer [13], prompt for new advances in HER2-targeted therapy for a population of patients with type 2 endometrial cancer [14,15]. In addition, there are few treatment options for other patients with advanced or recurrent endometrial cancer, but the prognosis of such patients remains poor. For women with advanced and recurrent uterine carcinosarcomas, first-line treatment remains combination chemotherapy with carboplatin and paclitaxel [16].

Sortilin (SORT1), which is also known as the neurotensin receptor 3 or 95-kDa RAP binding protein (Gp95), is a multitasking protein involved in numerous pathological processes such as cancer development, cardiovascular impairment, metabolic diseases, and depression [17,18]. SORT1 is an intrinsic transmembrane protein described as a scavenger receptor, which can be detected at the plasma membrane where it has been shown to bind and internalize several peptides and proteins, most notably neurotrophins [19,20]. The expression of SORT1 has previously been shown to be strongly increased in human malignancies including ovarian and endometrial cancers [21,22,23]. SORT1 is located within cells where it is involved in the trafficking of vesicles between intracellular compartments [18], and its siRNA-mediated gene silencing in an ovarian tumor cell line leads to increased apoptosis and diminished proliferation [21,22]. Increased levels of SORT1 expression have also been reported for other forms of cancers [24,25,26,27], and some studies have suggested that targeted inhibition of SORT1 expression and/or function could be useful for slowing the growth of tumors [21,24,26].

TH1902 is a peptide-drug conjugate with a payload of two docetaxel molecules ester-linked to a peptide (TH19P01) designed to recognize SORT1. Studies in breast and ovarian cancer cell lines have shown that TH1902 exploited SORT1’s ligand internalization functions and exerted potent antiproliferative and anti-migratory effects [28,29]. Within the cell, the docetaxel molecules are released from the conjugate and can then affect polymerization of microtubules leading to aberrant mitosis and apoptosis [28]. Intravenous administration of TH1902 to mice bearing xenografts of MDA-MB-231 breast cancer cells demonstrated a marked superiority of TH1902 over free docetaxel in preventing the growth and relapse of subcutaneous xenografts [28]. In a previous study, SORT1 was reported to have a role in vasculogenic mimicry (VM), and TH1902 was shown to inhibit in vitro VM in these cells and in the ES-2 ovarian cancer cell line [29].

In the current study, high levels of SORT1 expression were confirmed in ovarian cancer tissues and ovarian cancer-derived cell lines, and these findings were further extended to endometrial cancer tissues and respective cell lines. The effects of TH1902 and docetaxel on the growth of human ovarian and endometrial cancer cell lines were examined in vitro while the effects of these agents, alone or combined with carboplatin, on tumor growth were examined in vivo using xenografts from the same cell lines in mice.

## 2. Materials and Methods

### 2.1. Reagents, Cell Lines, and Antibodies

The preparation of TH1902 has been described elsewhere [28]. Neurotensin and the TH19P01 peptide (as well as a version with an additional C-terminal Cys) were synthesized and purified in-house with peptide purity > 95%. TH19P01-Alexa^488^ was obtained by first synthesizing the TH19P01 peptide with an additional Cys residue at the C-terminus, then linking Alexa Fluor^TM^ 488 C5 maleimide (#A10254 Thermo Fisher Scientific, Waltham, MA, USA) to that cysteine following the manufacturer’s instructions, followed by purification by reversed-phase HPLC. Lipofectamine 2000, Lysotracker Red and 4′,6-diamidino-2-phenylindole (DAPI) were from Thermo Fisher Scientific. Docetaxel was from Tecoland Corporation (Irvine, CA, USA). The siRNAs against a random scrambled sequence (siSCR; AllStar Negative Control siRNA, 1027281) and SORT1 (siSORT1; Hs_SORT_4 FlexiTube siRNA: SI00073640) were obtained from Qiagen (Toronto, ON, Canada). The tissue microarrays (#NBP2-30170 for normal tissues, #NBP2-30290 for ovarian cancers and #NBP2-30330 for diverse cancers) were purchased from Novus Biologicals (Toronto, ON, Canada). Respective and detailed clinical data of the tissue samples used can be found at the following vendors’ web site (https://www.novusbio.com/products/multi-organ-tissue-micro-array_nbp2-30170; https://www.novusbio.com/products/ovary-tissue-micro-array_nbp2-30290; https://www.novusbio.com/products/various-tissue-micro-array_nbp2-30330; all web sites accessed on 5 April 2022). All other reagents were from Sigma-Aldrich (Oakville, ON, Canada). The following human cell lines were purchased from the American Type Culture Collection (ATCC, Manassas, VA, USA): MDA-MB-231, ES-2, OVCAR-3, AN3-CA, HEC-1-A, HEC-1-B, SK-UT-1B and KLE. The SKOV3 human cell lines was purchased from Cell Biolabs (San Diego, CA, USA), and the A2780 human cell line was purchased from the European Collection of Authenticated Cell Cultures (ECACC, Porton Down, UK). The anti-SORT1 antibody (clone F11, EMD Millipore #MABN1792) used for IHC was a purified mouse monoclonal IgG1κ antibody which was raised against a recombinant protein corresponding to the extracellular domain of human SORT1. The anti-SORT1 antibody used for Western blots was a rabbit polyclonal antibody (#ab16640, Abcam, Cambridge, MA, USA).

### 2.2. Cell Line Characteristics and Culture Conditions

All cells were cultured according to the vendor’s instructions. For experiments, cells from frozen aliquots were seeded and subcultured for 5–10 passages. ES-2 is a CCC of the ovary, a subtype of EOC known for its relative resistance to standard platinum-based chemotherapy and poor prognosis. A2780 is an EOC cell line that was established from an ovarian endometroid adenocarcinoma tumor in an untreated patient. SKOV3 is a human EOC cell line resistant to tumor necrosis factor and to other cytotoxic drugs such as diphtheria toxin, cisplatin, and adriamycin. The AN3-CA cell line was derived from a metastatic lesion in the lymph node of a patient with an undifferentiated type 1 endometrial carcinoma harboring alterations in the PI3K/Akt pathway.

### 2.3. Animals

Animals were obtained from Charles River Laboratories, Inc. and allowed to acclimate for 5 days before experiments. Female CD-1 nude mice (*Crl:CD1-Foxn1^nu^*, 4–6 weeks old; ES-2) or athymic nude mice (*Crl:NU(NCr)-Foxn1^nu^*, 4–6 weeks old; SKOV3, A2780, AN3-CA) were used for the xenograft tumor models. Mice were maintained in a pathogen-free environment and handled in accordance with the Guidelines of the Canadian Council on Animal Care. Animal protocols were approved by the Institutional Animal Care and Use Committee of Université du Québec à Montréal (0620-C1R1-897-0621).

### 2.4. Fluorescence Measurement of TH19P01-Alexa^488^ Internalization

Cells were grown in 12-well plates in complete media for 24 h. Where required, cells were transiently transfected with 100 nM of siRNA (either a scrambled, control siRNA, or one that was directed against a particular mRNA) for 24 h using Lipofectamine 2000. Cells were next washed with PBS and then incubated in HBSS without phenol red for 2 h at 37 °C in the presence or absence of 200 nM Alexa^488^-labeled TH19P01 peptide. Alexa^488^-labeled TH19P01 peptide uptake was also measured with or without competition by an excess of unlabeled TH19P01 peptide (50 µM) or neurotensin (10 µM) during a 2 h uptake before washing with serum-free media. Cell fluorescence was evaluated in the FL1 channel using a C6 Accuri flow cytometer (BD Biosciences, Mississauga, ON, Canada).

### 2.5. Tissue Microarray Probing and Analysis

SORT1 expression was evaluated using commercial high-density tissue microarrays (TMAs) of different human subtypes of ovarian and endometrial cancers, as well as of healthy tissues, at the Institute for Research in Immunology and Cancer (IRIC; Montreal, QC, Canada). Immunostaining was performed as described previously by us [28]. An accredited pathologist, Dr. Louis Gaboury, performed analysis of the images acquired from the immunohistochemistry. SORT1 labeling was scored on a scale ranging from 0 to 3 as follows: 0, negative staining; 1, weak staining; 2, moderate staining; and 3, strong staining. The proportion of cells showing positive staining was recorded as follows: 1, none; 2, 10–50%; 3, 50–70%; and 4, 70–100%. The raw data were converted to an immunohistochemical score by multiplying the quantity and staining intensity scores. Therefore, the IHC score ranged from 0 to 12.

### 2.6. cDNA Measurements on Tissue cDNA Microarray

SORT1 gene expression in a TissueScan ovarian cancer cDNA array II (OriGene, #HORT102, Rockville, MD, USA) was quantified by qPCR using SsoFast EvaGreen^®^ Supermix (Bio-Rad) in a CFX Connect Real-Time PCR System (Bio-Rad, Mississauga, ON, Canada). The relative quantities of human *SORT1* were compared against a human *GAPDH* internal control and were measured by following a Ct (Cycle threshold) method employing an amplification plot (fluorescence signal vs. cycle number) and obtaining a cycle threshold. Primer pairs (*SORT1*, QT00073318; *GAPDH*, QT00079247) were from Qiagen. For each sample, the Ct value of the target gene and of *GAPDH* was calculated using the CFX ManagerTM software version 3.1 (Bio-Rad) and the normalized expression (ΔCt) was quantified. Detailed clinical data of the tissue samples used can be found at the vendors’ web site (https://www.origene.com/products/tissues/tissuescan, accessed on 5 April 2022).

### 2.7. Western Blot

Cell monolayers (MDA-MB-231, ES-2, OVCAR-3, AN3-CA, HEC-1-A, HEC-1-B, SK-UT-1B, KLE, SKOV3, and A2780) were homogenized in lysis buffer (150 mM NaCl, 10 mM Tris-HCl, pH 7.4, 1 mM EDTA, 1 mM EGTA, 0.5% (vol/vol) Nonidet P-40 and 1% (vol/vol) Triton X-100) supplemented with a complete protease inhibitor cocktail (Sigma-Aldrich, Oakville, ON, Canada). Cells were processed for electrophoresis and immunodetection with the anti-SORT1 primary antibody as described previously [28]. Signals were detected using chemiluminescence (Clarity Western ECL, Bio-Rad, Mississauga, ON, Canada).

### 2.8. Photomicrographs of Cells Labeled with DAPI, Lysotracker, or with Fluorescent Peptide

Cells plated on glass coverslips and grown to 70% confluence were incubated for 17 h with 0.5–1 µM TH19P01-Alexa^488^ peptide and 50–100 nM Lysotracker at 37 °C. Cells were washed with PBS, fixed for 15 min in 4% paraformaldehyde and then permeabilized (1% Triton X-100 in PBS for 5 min). After a final wash, cells were stained with DAPI (2 µg/mL; Invitrogen, Waltham, MA, USA) for 4 min, washed again and mounted onto slides using Prolong Gold antifade reagent. Photomicrographs were taken and digitalized by confocal microscopy (Nikon A1, Melville, NY, USA) and analyzed using NIH ImageJ Version 1.4.21 software.

### 2.9. Cell Line Growth Measurement

To assess the effects of docetaxel and TH1902 on ovarian and endometrial cancer cell proliferation, cells (ES-2: 3000 cells/well, SKOV3: 1000 cells/well, A2780: 7000 cells/well, AN3-CA: 7000 cells/well) were first seeded in 96-well plates (Perkin Elmer, Guelph, ON, Canada), then treated with various concentrations of drugs in complete cell culture medium. After 72 h of incubation for ES-2, A2780 and AN3-CA cells, or 96 h for SKOV3 cells, cell proliferation was measured using the 3-(4,5-dimethylthiazol-2-yl)-2,5-diphenyltetrazolium bromide (MTT) as described previously [28,30]. Analyses were made in quadruplicate for each condition.

### 2.10. Cell Apoptosis Assay

AnnexinV/PI staining was performed using an Apoptosis Detection Kit according to the manufacturer’s instructions (BD Pharmingen, San Diego, CA, USA). Briefly, after treatment, ES-2 and SKOV3 cells were harvested, resuspended in a staining solution of 100 µL of 1X binding buffer containing 5 µL of AnnexinV-FITC and 5 µL of PI. Cells were incubated for 15 min at room temperature in the dark, and the number of apoptotic cells were acquired and analyzed using a BD Accuri C6 flow cytometer.

### 2.11. In Vivo Therapeutic Efficacy Assessment of Docetaxel and TH1902 Using Xenograft Models

Tumor xenografts were established by subcutaneous inoculation of 5 × 10^6^ cells (ES-2) or 7 × 10^6^ cells (SKOV3) in 150 µL of HBSS; other xenografts were established by subcutaneous inoculation of 5 × 10^6^ cells (A2780) or 2 × 10^6^ cells (AN3-CA) in 150 µL of HBSS/Matrigel (50:50). All cells were injected into the right flanks of immunodeficient mice under light isoflurane anesthesia. The injection schedules and doses of injected substances are detailed in the figures. Briefly, when palpable ES-2, SKOV3 or AN3-CA tumors reached a volume of ~100 mm^3^, mice were treated weekly with either vehicle, docetaxel (3.75 or 15 mg/kg) or TH1902 (8.75 or 35 mg/kg) at equivalent docetaxel amounts via intravenous (IV) tail vein injection. Note that three cycles of 15 mg/kg docetaxel is the maximum tolerated dose in mice [31] and that 35 mg/kg TH1902 contains the same quantity of docetaxel (bis-linked to the peptide). In A2780 xenografts, when palpable tumors reached a volume of ~250 mm^3^, mice were treated with either vehicle, paclitaxel (10 mg/kg, IV), carboplatin (40 mg/kg, intraperitoneal (IP)), docetaxel (10 mg/kg, IV) or TH1902 (23 mg/kg, IV) bi-weekly as single agents or once on Day-0 as combinations with carboplatin. For combination treatments, both taxanes and TH1902 were injected 1 h following carboplatin administrations at the doses previously described. Injectable solutions were prepared as follows: TH1902 was solubilized at 10 mg/mL in 10% Tween-80 in D5W (pH 4.3, *w*/*v*) then further diluted with D5W; docetaxel was solubilized at 25 mg/mL in ethanol and Tween-80 (50/50, *v*/*v*) then further diluted with D5W; paclitaxel was solubilized at 25 mg/mL in ethanol and Cremophor EL (50/50, *v*/*v*) then further diluted in saline; carboplatin was solubilized at 50 mg/mL in water then further diluted in a solution of 30% PEG400 and 5% Tween-80 in D5W (*w*/*v*); vehicle composition and preparation mimicked highest dose of TH1902 preparation. Tumor growth was monitored by two-dimensional measurements taken with an electronic caliper and tumor volume was calculated according to the following formula: tumor volume (mm^3^) = π/6 × length × width^2^. Data are expressed as mean ± SEM and were analyzed using GraphPad Prism software.

### 2.12. Statistical Data Analysis

Data are expressed as means ± standard error of the mean (SEM) or standard deviation (SD) as indicated in the figure legends. Statistical analysis was done using *t*-test for comparing two samples, whereas analysis by one-way ANOVA followed by Dunnett’s multiple comparisons was employed for three or more samples. A value of *p* < 0.05 was considered significant and one or more asterisks (*) identifies the level of such significance in the figures.

## 3. Results

### 3.1. SORT1 Expression in Ovarian and Endometrial Tissues and Tumors

**Figure 1:**
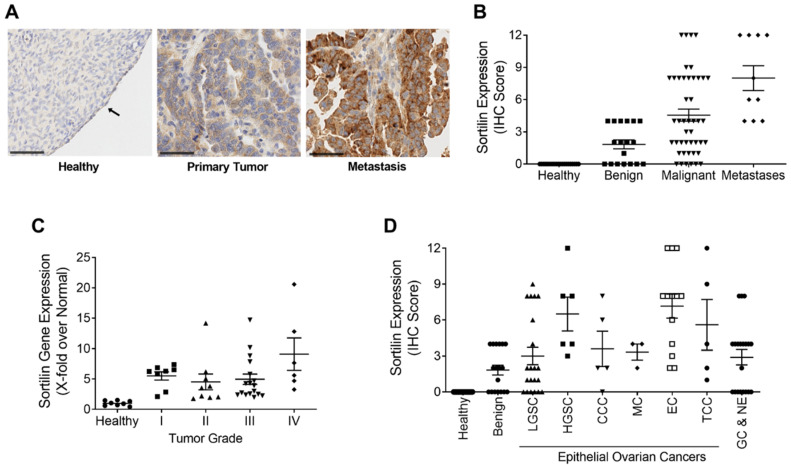
Expression of SORT1 in ovarian healthy and tumoral tissues. Immunohistochemical staining of SORT1 in tissue microarrays (TMAs) was used to measure the level of SORT1 expression in healthy tissues and in ovarian tumors. With the immunohistochemical (IHC) scoring system used, the level of expressed SORT1 ranged from 0 to a maximum of 12. (**A**) Representative SORT1 staining by IHC in ovarian healthy, primary and metastases biopsies. Nuclei are stained blue with hematoxylin. Arrows point to ovarian healthy epithelia. Black bars represent 50 µm. (**B**) IHC scores from healthy tissues (*n* = 20), benign tumors (*n* = 18), malignant tumors (*n* = 45) and metastases (*n* = 10). (**C**) RT-qPCR was used to quantify the transcript levels of *SORT1* in healthy and cancerous ovarian tissues. The *SORT1* transcript levels in tumor samples were segregated according to tumor grade and then compared to the values from healthy ovarian tissues. *n* = 8 for healthy and for Grade I tumor samples, *n* = 9 for Grade II, *n* = 17 for Grade III and *n* = 7 for Grade IV tumor samples. (**D**) The mean IHC scores for SORT1 is shown for healthy ovarian tissue (*n* = 20), benign tumors (*n* = 18), LGSC (*n* = 21), HGSC (*n* = 6), CCC (*n* = 5), MC (*n* = 3), EC (*n* = 13), TCC (*n* = 5) and GC and NE (*n* = 20). All scatter plots include lines showing the means ± SEM, and where each point represents an individual tissue sample.

**Figure 2:**
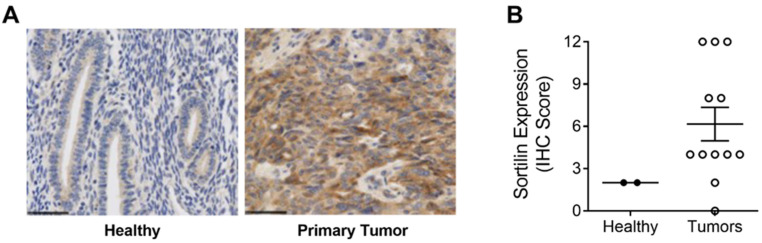
Expression of SORT1 in endometrial healthy tissues and endometrial tumor biopsies. Immunohistochemical staining of SORT1 in tissue microarrays (TMAs) was used to measure the level of SORT1 expression in healthy tissues and in endometrial tumors. (**A**) Representative SORT1 staining by IHC in endometrial healthy tissue and endometrial tumor biopsies. Nuclei are stained blue with hematoxylin. Black bars represent 50 µm. (**B**) SORT1 IHC scores between healthy endometrial tissues (*n* = 2) and endometrial cancerous tissues (*n* = 12). Scatter plot include lines showing the means ± SEM, and where each point represents an individual tissue sample.

Commercial tissue microarrays (TMAs) containing human tissues and clinically annotated tumors were probed for SORT1 expression using IHC. SORT1 expression was found higher in ovarian primary tumors and metastasis, than in healthy ovarian tissues where SORT1 was below detectable levels as was shown elsewhere (Figure 1A) [22]. A marked increase was observed in SORT1 levels between healthy tissue and either malignant primary ovarian tumors or metastases from ovarian tumors (Figure 1B). Ovarian tumors consist of three main types (epithelial, germ-cell and sex-cord-stromal), of which the epithelial type represents 95% of the known cases of ovarian tumors [32]. Further, ovarian carcinosarcomas (OCS), known as mixed malignant Müllerian tumors are challenging histologic subtypes accounting for only 1–4% of all ovarian cancer. Histologically, carcinosarcomas are composed of an epithelial as well as a sarcomatous component. OCS appear to follow a distinct natural history compared to other more common epithelial carcinomas. Their prognosis is dismal, and most patients relapse within one year after completion of initial treatment [33]. Within the samples tested here using TMAs, SORT1 expression was observed in about 60% of the benign tumor samples as well as in the germ cell and non-epithelial tumors. Its gene expression levels increased in most epithelial tumors and appeared to vary according to ovarian tumor grade (Figure 1C). IHC scoring was also performed with regard to ovarian cancer subtypes and compared to the very low levels of SORT1 found in healthy tissues. SORT1 expression increased in 11/21 (52%) cores of LGSC, in 6/6 (100%) cores of HGSC, in 4/5 cores (80%) of CCC in 3/3 cores (100%) of MC in 13/13 cores (100%) of EC, and in 4/5 cores (80%) of TCC (Figure 1D). Overall, SORT1 expression was higher in about 77% of all epithelial ovarian tumors tested, the percentage increased to 94% outside of LGSC. In addition, SORT1 was detected in 60% (12/20 cores) of GC and NE tumors. IHC SORT1 staining was also performed in TMAs of endometrial cancers (Figure 2). Within the samples tested here, increased SORT1 staining was observed in endometrial primary tumors compared to healthy endometrial tissue (Figure 2A). As indicated by the IHC scores, 10 out of 12 (83%) tumors tested showed higher expression levels of SORT1 than in normal endometrial tissue (Figure 2B). In light of such preliminary screen, the suitability of SORT1 as a target for a peptide-docetaxel treatment or as a biomarker for patient screening will be evaluated in the clinical setting using a validated assay, but for the moment should be considered and interpreted with caution.

### 3.2. SORT1 Expression in Gynecological Cancer Cell Lines

**Figure 3:**
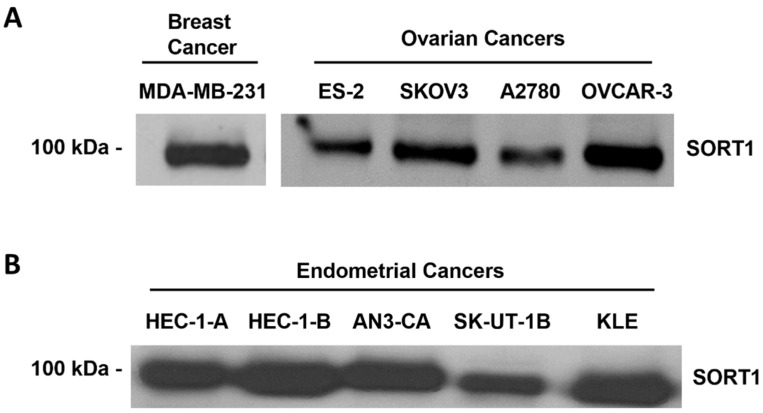
SORT1 expression in ovarian and endometrial cancer cell lines. Cell lysates were harvested from different commercially available human ovarian, endometrial and breast cancer-derived cell lines. SORT1 protein expression was assessed using Western blotting in (**A**) ovarian and TNBC-derived cells, and (**B**) endometrial cancer cells.

SORT1 expression was next examined in a series of commercially available cell lines derived from human ovarian and endometrial cancers (Figure 3). High levels of SORT1 were detected in the ES-2, SKOV3, A2780, and OVCAR-3 ovarian cancer cell lines (Figure 3A; Figure S4 in the Supplementary Materials Section). Their level of expression was comparable to that seen in the TNBC-derived MDA-MB-231 cell line, which is noted for its high level of SORT1 expression [28]. The SKOV3, OVCAR-3 and A2780 cell lines had been previously shown to express SORT1 [22]. High expression of SORT1 was also observed in HEC-1-A, HEC-1-B, AN3-CA, SK-UT-1B and KLE endometrial cancer cell lines (Figure 3B; Figure S5 in the Supplementary Materials Section). This suggests that the above ovarian and endometrial cancer cell lines express sufficient SORT1 as to enable experimentation involving this protein.

### 3.3. SORT1 Mediates TH19P01 Internalization to Lysosomes

**Figure 4:**
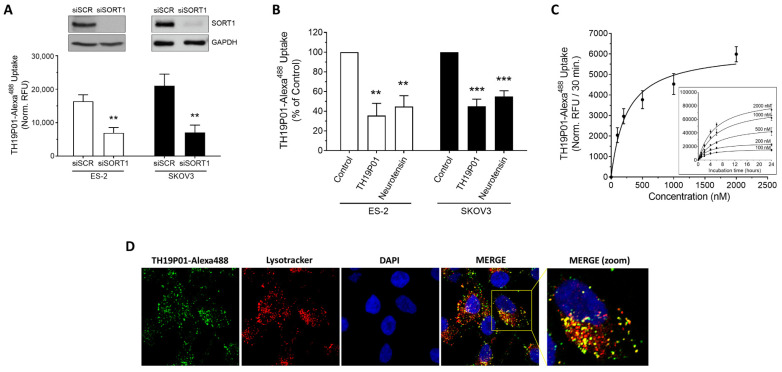
Internalization of TH19P01-Alexa^488^ peptide by SORT1 in ovarian cancer cell lines. (**A**) Uptake of TH19P01-Alexa^488^ was examined into ES-2 and SKOV3 ovarian cancer cell lines after prior incubation with siRNA, either a scrambled control (siSCR) or siRNA directed against SORT1 (siSORT1). t-tests were used to assess the significance of the difference between fluorescent uptakes (** *p* < 0.01). Representative SORT1 protein detection by Western blot (*n* = 3). Data represent the mean of net RFU ± SEM (*n* = 3 to 4). (**B**) Uptake of TH19P01-Alexa^488^ into two ovarian cancer cell lines was measured in the presence of excess concentrations of substrates known to bind to SORT1 (50 µM TH19P01 and 10 µM neurotensin). Fluorescent uptake was compared between cells treated with vehicle (Control) and those treated with the competing ligands using one-way ANOVA and Dunnett’s multiple comparison test (**, *p* < 0.01; *** *p* < 0.001). Data represent the percentages to control net RFU mean ± SEM (*n* = 4) (**C**) Time-course of TH19P01-Alexa^488^ uptake in SORT1-positive ES-2 cells with increasing concentrations (inner curves set). Uptake values at 30 min was next plotted according to TH19P01-Alexa^488^ concentrations to calculate kinetic parameters using a one site-specific binding analysis with the GraphPad Prism Software (outer curve set). Results are expressed as normalized RFU according to measurements of control wells. Data represent the mean and SD (*n* = 3). (**D**) ES-2 ovarian cancer cells were incubated in the presence of TH19P01-Alexa^488^ (green) and Lysotracker^TM^ (red). The cells were then washed and fixed, then nuclei stained with DAPI (blue). Photomicrographs were taken in separate channels for each of the fluorophores used and merged. Colocalized pixels are shown in yellow in the merged panels (*n* = 3). Magnification = 60×.

Once SORT1 has recognized the TH19P01 peptide and internalized it, it could conceivably be conveyed to several subcellular compartments, as SORT1 is known to be involved in vesicle trafficking [34]. To first confirm the requirement of SORT1 for peptide internalization, siRNA-mediated silencing of *SORT1* was performed in ES-2 and SKOV3 ovarian cancer cells and TH19P01-Alexa^488^ uptake found significantly decreased by 58% and 67%, respectively (Figure 4A; Figure S6 in the Supplementary Materials Section). Uptake was also found reduced in those same cell lines when concomitantly incubated with either excess unlabeled TH19P01, or a known ligand of SORT1, namely neurotensin (Figure 4B). Uptake of THP1901-Alexa^488^ was further measured over time in ES-2 cells as a function of fluorescent peptide concentration and found saturable (Figure 4C). The KD and Bmax constants of TH19P01 were extracted from the plots and calculated at 248 nM and 6190 RFU/30 min, respectively (Figure 4C). Subcellular compartmentation of the TH19P01-Alexa^488^ was also examined in ES-2 ovarian cancer cells in the presence of Lysotracker^TM^, which is used for staining acidic organelles and to label lysosomes. Colocalization of the fluorescent TH19P01-Alexa^488^ with Lysotracker labeling indicates that, once internalized, the fluorescent peptide reaches the lysosomes (Figure 4D).

### 3.4. In Vitro TH1902 Anticancer Activity against Ovarian Tumor Cell Lines

**Figure 5:**
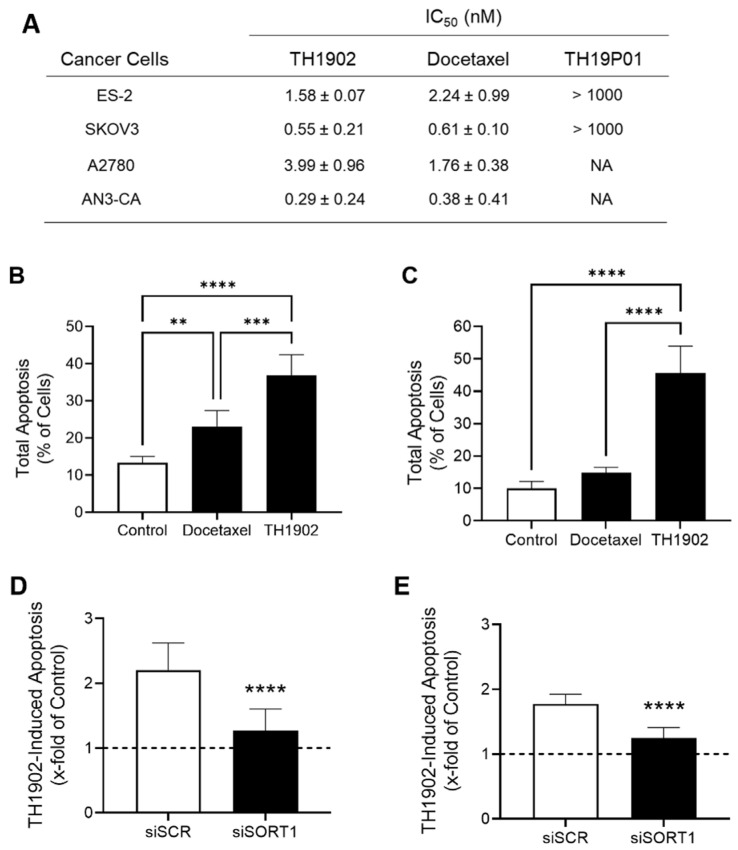
Effects of TH1902 on ovarian and endometrial cancer cells on proliferation and cell death. (**A**) The anti-proliferative activities of docetaxel, TH1902 and TH19P01 was determined using MTT assays in ES-2, SKOV3, A2780 and AN3-CA cancer cells as described in the Methods section. IC_50_ values are presented as mean ± SD (*n* = 3 to 5). (**B**) ES-2 and (**C**) SKOV3 cells were treated for 5 h with 2 µM docetaxel, or TH1902. Cells were then harvested, and the extent of apoptotic cell death determined by flow cytometry following staining with AnnexinV-FITC and propidium iodide (PI). Data are represented as mean ± SD (*n* = 3). TH1902-mediated apoptosis following transient *SORT1* gene silencing was investigated in (**D**) ES-2 and (**E**) SKOV3 as described in the Methods section. Dotted lines indicate the apoptosis induction level in untreated control cells. All data are represented as mean ± SD (*n* = 3), and statistical analysis was performed using one-way ANOVA with Dunnet’s multiple comparison test (**B**,**C**) or Student’s unpaired t test (**D**,**E**) (**, *p* < 0.01; *** *p* < 0.001; **** *p* < 0.0001).

Since SORT1 is expressed in ovarian and endometrial tumors as well as in cell lines derived from these tumors, and since SORT1 binds and internalizes the TH19P01 peptide, it was next investigated whether TH1902 could be used to treat such tumors. The ovarian cancer cell lines ES-2, SKOV3, A2780 and AN3-CA were used to determine whether TH1902 could inhibit their proliferation in vitro. Cells were thus exposed to various concentrations of TH1902 or docetaxel, and the effects on cell proliferation were measured using the MTT detection assay. It is apparent that both reagents exerted a cytotoxic effect with IC_50_ values of TH1902 comparable to that of docetaxel at low nM concentrations in the four cell lines tested (Figure 5A). This supports the rationale that conjugated docetaxel can be released from TH1902 and exert its anti-proliferative effect inside the targeted cancer cells. Parallel experiment showed that cell proliferation was unaffected by the free peptide (TH19P01) at concentrations up to 1000 nM (Figure 5A). The effect of Docetaxel and TH1902 on apoptosis induction in ES-2 and SKOV3 cells was also tested following 5 h of treatment (Figure 5B,C). The results show that TH1902 induces more efficiently apoptosis in both cell lines when compared to docetaxel especially in SKOV3 cells. Overall, this suggests that receptor-mediated events appear to account for the increased effects of TH1902 within such a short time frame. To confirm the implication of SORT1 in TH1902 internalization and apoptosis induction, SORT1 was transiently silenced in ES-2 and SKOV3 cells. SORT1 repression significantly reduced by 78% (ES-2) and 68% (SKOV3) the apoptosis induction by TH1902 in both cell lines (Figure 5D,E, respectively). This result further supports a SORT1 receptor-mediated internalization mechanism for TH1902. Overall, these data confirm the cytotoxic activity of TH1902 against ES-2 and SKOV3 ovarian cancer cells, just as was previously shown against the SORT1-expressing TNBC cell line MDA-MB-231 [25].

### 3.5. TH1902 and Docetaxel Activity In Vivo against ES-2 and SKOV3 Ovarian Tumor Xenografts

**Figure 6:**
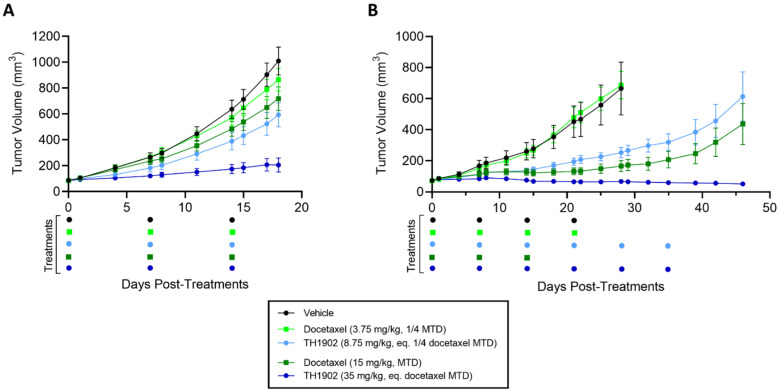
Effects of docetaxel and TH1902 on ES-2 and SKOV3 ovarian cancer xenografts. Comparative efficacy of docetaxel and TH1902 on ovarian cancer xenografts tumor growth. Immunodeficient mice bearing subcutaneous ES-2 (**A**) or SKOV3 (**B**) tumors were intravenously injected weekly with either vehicle, docetaxel (at its MTD and quarter MTD doses; 15 and 3.75 mg/kg, respectively), or TH1902 (at a dosage which contains a quantity of bound docetaxel equal to the docetaxel doses; 35 and 8.75 mg/kg, respectively), as described in the methods section. Mice in the ES-2 study were euthanized at vehicle group endpoint (Day 18), whereas indicated groups in SKOV3 study were monitored for a prolonged period up to Day 46 post start of treatments. Three cycles of docetaxel at 15 mg/kg were considered MTD. Colored dots below the abscissa indicate drug treatment events for all groups. Data are represented as mean ± SEM (*n* = 6 mice/group).

Two ovarian tumor xenograft models (ES-2 and SKOV3) were used to test the anticancer activities of TH1902 and of docetaxel. These mice were treated, at a dosage equivalent in terms of docetaxel content, with either low doses (3.75 vs. 8.75 mg/kg; a quarter of docetaxel MTD) or high doses (15 vs. 35 mg/kg; docetaxel MTD) of docetaxel and TH1902, respectively. In both ovarian tumor xenograft models, TH1902-treated mice showed stronger tumor growth inhibitions over matching doses of unconjugated docetaxel (Figure 6). In the ES-2 study, mice treated with low and high doses of TH1902 significantly inhibited the growth of tumors by, respectively, 45% and 87%, whereas both docetaxel groups, at equivalent docetaxel content, produced little effect (Figure 6A and Table 1). Low doses of docetaxel and low and high doses of TH1902 were well-tolerated with slight weight gain, whereas three weekly cycles of treatments with high doses of docetaxel (the MTD for this agent in mice) produced a small weight loss when compared to initial mice weights (Figure S1 in the Supplementary Materials Section).

In the SKOV3 study, mice treated with low and high doses of TH1902 showed significant inhibitions of tumor growth where only high dose of docetaxel produced this effect (Figure 6B). When compared to vehicle endpoint (Day 28), low dose of TH1902 produced similar tumor growth inhibitions when compared to high dose of docetaxel (69% vs. 84%, respectively) while high dose of TH1902 induced stronger inhibition with slight regression of tumors (Table 1). Moreover, mice treated with high dose of TH1902 showed prolonged inhibitory effect (up to Day 46) where docetaxel-treated mice could not sustain this effect, which is marked by tumor regrowth (Figure 6B). As mentioned above, three weekly cycles of 15 mg/kg is the maximum tolerated dose (MTD) for docetaxel in mice. When treated at equivalent docetaxel MTD doses, docetaxel-treated mice receiving three cycles of treatments showed severe weight loss (>10% loss) in contrast to TH1902-treated mice which displayed minimal weight loss effects even after receiving twice the weekly cycles (three vs. six cycles) of treatment (Supplementary Materials Figure S1B). Furthermore, we previously demonstrated the absence of hematotoxicity of TH1902 at this dose in mice where no decrease in neutrophils counts was observed after six cycles of TH1902 in contrast to docetaxel at an equivalent dose [28]. The increased tolerability of TH1902 over docetaxel allowed for prolonged treatments.

### 3.6. TH1902 and Docetaxel Activity In Vivo against AN3-CA Endometrial Tumor Xenografts

**Figure 7:**
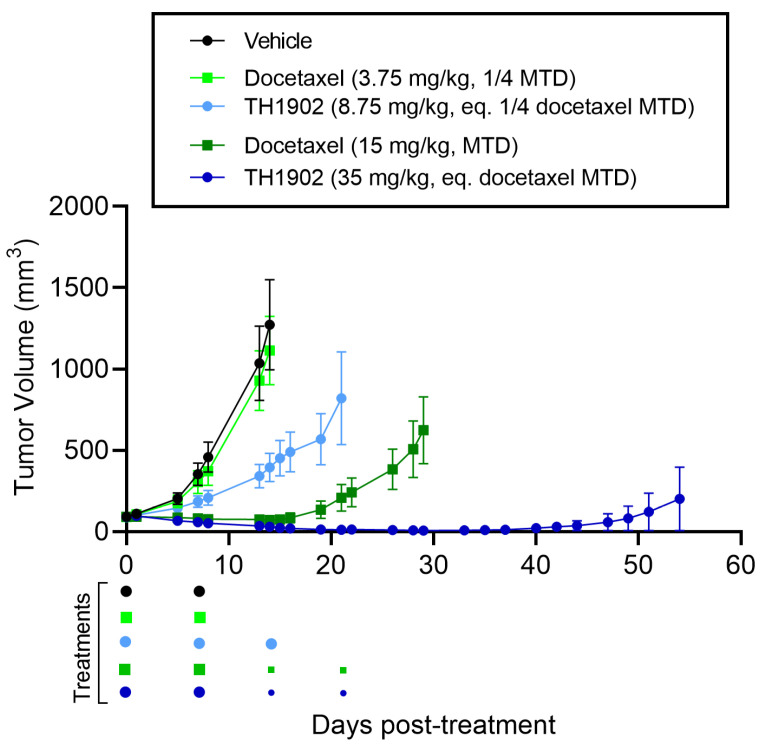
Effects of docetaxel and TH1902 on AN3-CA endometrial cancer xenografts. Comparative efficacy of docetaxel and TH1902 on endometrial cancer xenografts tumor growth. Immunodeficient mice bearing subcutaneous AN3-CA tumors were intravenously injected weekly with either vehicle, docetaxel (at its MTD and quarter MTD doses; 15 and 3.75 mg/kg, respectively), or TH1902 (at a dosage which contains a quantity of bound docetaxel equal to the docetaxel doses; 35 and 8.75 mg/kg, respectively). Low dose docetaxel group was euthanized at vehicle group endpoint (Day 14) whereas TH1902 low dose, docetaxel and TH1902 high doses were monitored for a prolonged period (Days 21, 29 and 54 post start of treatments, respectively) as described in the methods section. Colored dots below the abscissa indicate drug treatments for all groups, high doses of docetaxel and TH1902 were cut by half (7.5 and 17.5 mg/kg, respectively) for the third and fourth treatments because of docetaxel group weight loss (small dots). Data are represented as mean ± SEM (*n* = 6 mice/group).

For mice bearing endometrial tumor xenografts, a similar dosage regimen to that for ovarian tumor xenografts was used as described previously for both docetaxel and TH1902. The growth of AN3-CA tumors was inhibited by both dosages of TH1902, whereas only a high dose of docetaxel could produce this effect (Figure 7). When compared to vehicle endpoint, low dose of TH1902 significantly inhibited the growth of AN3-CA tumors by 74%, whereas both high doses of docetaxel and TH1902 induced tumor regressions (Table 1). Interestingly, a large portion of tumors (5 out of 6) within the high-dose TH1902 group were unmeasurable or remained in regression for a prolonged period (up to Day 54), whereas tumors in the equivalent docetaxel dose were unresponsive and regrew before the end of treatments (Figure 7). The two high doses were diminished by half for the final two treatments due to weight loss in the animals receiving high-dose docetaxel (Figure S2 in the Supplementary Materials Section). Rapid weight loss was associated with administration of high dosage docetaxel, but this was reversed when the dosage was halved. In contrast, only mild weight loss was observed in animals administered with high-dosage TH1902.

### 3.7. Combined TH1902 and Carboplatin Activity In Vivo against A2780 Ovarian Tumor Xenografts

**Figure 8:**
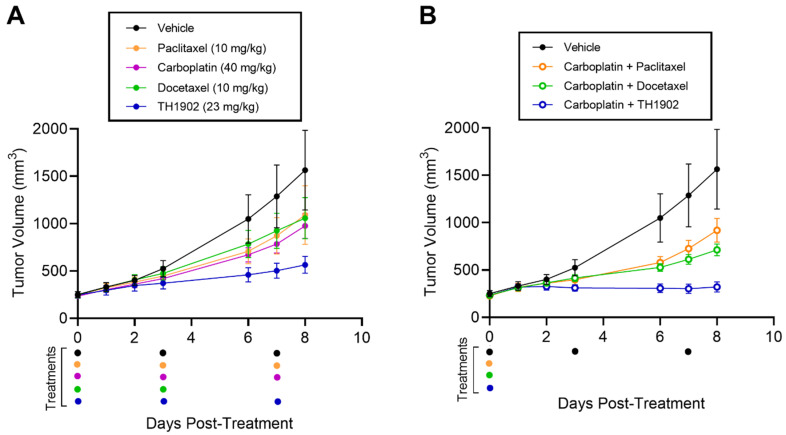
Effects of TH1902 and carboplatin combinations on A2780 ovarian cancer xenografts. A2780 cells were subcutaneously implanted in the flanks of athymic nude mice. Tumor volume measurements following bi-weekly administrations of (**A**) vehicle, docetaxel, TH1902, paclitaxel or carboplatin as single agents, or (**B**) administered once in combination using the same dosage as described in the methods section. Colored dots below indicate drug treatments for all groups. Data are represented as mean ± SEM (*n* = 5 mice/group).

Combination chemotherapy is commonly employed in the clinical treatment of ovarian cancer [35]. As a preliminary attempt at modeling TH1902 in combination chemotherapy, immunodeficient mice were implanted with subcutaneous xenografts of the A2780 ovarian cancer cell line. Treatments were initiated with the administration of single agents (bi-weekly) or in combination with carboplatin (single injection), as described in the methods section. TH1902-containing groups showed stronger tumor growth inhibition when injected as single agent (Figure 8A) or in combination with carboplatin (Figure 8B) compared to the other groups tested. In fact, when administered as a single agent, paclitaxel, docetaxel or carboplatin alone inhibited the growth of A2780 tumors by about 40% (36%, 43%, 38%, respectively), whereas TH1902 exhibited a 76% inhibition of tumor growth (Table 2). Moreover, a single injection of TH1902 in combination with carboplatin showed significant reduction in tumor growth (93%) compared to the standard of care combinations of carboplatin with either paclitaxel (47%) or docetaxel (63%). Only the administration of docetaxel for three cycles caused a significant body weight loss (Figure S3 in the Supplementary Materials Section).

## 4. Discussion

The current study highlights the TH1902 anticancer properties, a new PDC against gynecological cancers. Its in vitro anticancer properties as well as efficacy in ovarian and endometrial xenografts have been investigated. Given that the great majority of gynecological cancers still pose a considerable threat to women’s health, the current receptor-mediated chemotherapy therefore provides novel preclinical evidence for their treatment [1,36]. These cancers are characterized by poor long-term prognoses, producing death in half of the patients overall within five years [36]. This is primarily a consequence of diagnosis occurring at a late stage of the disease as well as by the development of drug resistance to the chemotherapeutic treatments commonly used, resulting in relapse [2].

The development of novel and highly effective anticancer drugs requires strategies that increase selective drug delivery to the tumor site and reduce side effects [37]. When designing anticancer peptide-drug conjugates, identification of differentially expressed cancer cell surface receptors therefore becomes of high interest in any targeted cancer therapy approach, as this provides the rationale that allows for selective tumor targeting. Here, through IHC scoring and Western blotting, high expression of SORT1 was observed in ovarian and endometrial metastatic and malignant tumor tissues as well as of corresponding cancer cell lines, in comparison to the respective healthy tissue. It therefore becomes reasonable to design treatment modalities that target SORT1 and which exploit SORT1 endocytic properties [28,29,34]. Indeed, results of the TMAs analysis established that high SORT1 expression is more tightly linked to EOC than to non-epithelial tumors. From the clinically annotated epithelial tumors screened herein, the majority exhibited increased SORT1 expression. Therefore, the evaluation of SORT1 expression may eventually help to better define the patient population that would be the most likely to benefit from TH1902 treatment. Thus, developing more specific and effective therapeutics is a prerequisite for significant improved outcomes in those cancer patients bearing SORT1-positive gynecological tumors.

In this study, the uptake of a fluorescently labeled peptide, used to generate TH1902, was first assessed to confirm the requirement of SORT1 for its internalization in SORT1-expressing ovarian and endometrial cancer cells. Next, the in vivo anticancer activity of the peptide-drug conjugate TH1902 was confirmed in xenograft models. The major goal of this study is to create and investigate new ovarian cancer treatments using targeted SORT1 receptor-mediated anticancer therapy. Overall, the results suggest that our peptide-drug conjugation could bring anticancer drugs, such as docetaxel, into tumors that overexpress SORT1. Interestingly, there is currently a trend toward tumors expressing SORT1 among HER2-positive tumors (77%) than among HER2-negative tumors (63%), whereas 59% of triple-negative breast cancers were positive [24]. In addition, recently reported in silico analysis of *SORT1* gene expression on the survival of TNBC patients confirmed that high *SORT1* gene expression is associated with a poor prognosis for TNBC patients in advanced 3 and 4 stages [28]. This further supports SORT1-positive cancers as therapeutic targets.

Taxanes, mainly paclitaxel and docetaxel, are well-known microtubule stabilizers used in various cancer including ovarian and endometrial cancer first-line therapies in the last thirty years [38,39]. Even though both of these taxanes significantly enhance the overall survival rate of cancer patients, very poor water solubility and the occurrence of severe side effects can be serious limitations to their use [38]. Here, the new chemical entity TH1902 is distinct from the docetaxel molecule as it selectively exploits SORT1’s internalization properties to trigger cell apoptosis in ES-2 and SKOV3 cells. Such process can be blocked through siRNA-mediated *SORT1* silencing indicating that the presence of the SORT1 protein is essential to the recognition, internalization, and pro-apoptotic processes of this peptide-drug conjugate. The internalization of fluorescent TH19P01 confirmed such mechanism as it was also blocked by excess quantities of TH19P01 or neurotensin, a known ligand for SORT1, showing that TH19P01 competes for the binding site(s) on SORT1. This supports a model where the peptide recognizes SORT1 and is internalized most probably through similar vesicular trafficking mechanisms as SORT1 does with its other peptides/proteins ligands. These vesicles are believed to be destined for the lysosomes, as was seen above with the fluorescently labeled TH19P01 in ES-2 ovarian cancer cells where it colocalized with the Lysotracker dye.

TH19P01 conjugation technology is very flexible and may have a broad range of therapeutic applications. According to the molecular rationale described above, this peptide-drug conjugation approach further presents several advantages over other approaches, such as those using cell-penetrating peptides (CPP) [40,41,42]. CPP’s ability to cross the cell membrane opened up, 20 years ago, avenues for drug delivery. Their lack of specificity and molecular cellular uptake mechanisms, however, remain to be better established [40,41,42]. Here, targeting SORT1 appears more specific than CPP for the delivery of anticancer drugs. Another approach currently developed in the oncology field and aimed at bringing cytotoxic anticancer drugs to specific cancer cells uses antibody drug conjugates (ADC). In ADC, highly cytotoxic molecules or toxins are incorporated onto monoclonal antibodies (mAb), which recognize specific antigens expressed on cancer cells in order to bring, in their close vicinity, a given bioactive agent [43]. Compared to antibodies, PDC such as TH1902 may offer many advantages in terms of intracellular uptake, tumor penetration, conjugation chemistry and manufacturing [44]. In comparison to the bulk weight or size of mAb carriers, the peptide carriers have the advantage of overcoming the interstitial tumor pressure allowing them to better reach the tumor interior [44,45]. Whereas the molecular structure of antibodies is standard—antibody unit with different immunoglobulin isotypes—peptides, on the other hand, offer versatile linear or cyclic structures [44]. Finally, our peptide conjugation platform, coupled to targeted receptor-mediated intracellular delivery, offers significantly increased product reproducibility, precise carrier stoichiometry, high loading efficiency, and potent agent efficacy.

After recovery from surgery, in patients with stage III and IV ovarian cancers or with recurrent diseases, combination chemotherapies are given. The combination used most often is carboplatin (or cisplatin) and a taxane, such as paclitaxel (Taxol) [46]. The targeted drug bevacizumab (Avastin) might be given along with chemotherapy as well in recurrent ovarian cancer patients [47,48]. The differences in response to docetaxel and TH1902 at equivalent low and high docetaxel doses are quite evident between the three ovarian xenograft models of ES-2, SKOV3 and A2780 cells as well as in the AN3-CA endometrial tumor xenograft model. In addition, rapid weight loss was used here, as an indication of deleterious side effects, and this was associated with docetaxel in some cases but not with TH1902. For these reasons, TH1902 appears superior to docetaxel for chemotherapeutic treatment of SORT1-expressing ovarian and endometrial tumors. Interestingly, the combination of TH1902 and carboplatin appeared to be more efficacious than the two other taxane-carboplatin combinations tested, and which are considered part of standard of care treatments [5,16].

Treatment of patients with aggressive endometrial cancers, the most common gynecological cancer in developed countries, will require novel therapeutic development [13]. Most patients with endometrial cancer have an early disease and favorable prognosis. However, a significant proportion of endometrial cancers, which mainly comprises high-grade or type 2 endometrial cancer such as HGSC, CCC, and EC, shows advanced/recurrent disease and dismal prognosis. Interestingly in the current study, increased SORT1 expression level was found in the clinically annotated samples tested in comparison to healthy tissue. Furthermore, TH1902 showed stronger tumor growth inhibition and caused long-term regression in mice bearing AN3-CA xenografts tumors. Among the molecularly targeted agents developed for patients with endometrial cancer, human epidermal growth factor receptor 2 (HER2) targeted therapy for patients with type 2 endometrial cancer has demonstrated practice-changing efficacy [13]. In this study, we uncovered the properties of TH1902, a new peptide-drug conjugate as receptor-mediated chemotherapy in SORT1-positive endometrial cancers.

## 5. Conclusions

In conclusion, these preclinical data on TH1902 suggest that this new SORT1 receptor-mediated anticancer drug conjugate could increase the efficacy of anticancer drugs with potentially less side effects. The fact that the scavenger receptor SORT1 is overexpressed in various types of ovarian, endometrial, and other types of cancers, such as breast cancer, further suggests that exploiting this receptor’s internalization functions could be used in other clinical indications. Importantly, TH1902 alone or in combination with carboplatin demonstrated better efficacy than taxanes or carboplatin-taxane based therapies for the treatment of both ovarian and endometrial cancers.

## Figures and Tables

**Table 1:** Tumor progression following administration of docetaxel or TH1902 in ovarian and endometrial cancer xenograft models.

Cancer Indications	Xenograft Models	Groups	Doses (mg/kg)	Tumor Progression ^a^	
				Size Increase (x-Fold Day-0)	Significance ^b^ (*p* Values)
**Ovarian cancer**	ES-2	Vehicle	0	11.84	na
		Docetaxel	3.75	10.08	ns
			15	8.31	ns
		TH1902	8.75	6.70	0.007
			35	2.37	<0.0001
	SKOV3	Vehicle	0	8.63	na
		Docetaxel	3.75	9.70	ns
			15	2.25	0.001
		TH1902	8.75	3.69	0.007
			35	0.94	0.0002
**Endometrial cancer**	AN3-CA	Vehicle	0	13.27	na
		Docetaxel	3.75	12.07	ns
			15	0.79	<0.0001
		TH1902	8.75	4.59	0.002
			35	0.35	<0.0001

^a^ Tumor size progression at vehicle group endpoints compared to start of treatments (Day 0). Ratios > 1 indicate tumor progression whereas ratios < 1 indicate tumor regression. ^b^ Significance between tumor progression of individual group vs. respective vehicle group endpoints using one-way ANOVA with Dunnett’s multiple comparisons test, na: not applicable, ns: not significant.

**Table 2:** Tumor progression following administration of chemotherapeutics as single agent or in combination with carboplatin in A2780 ovarian cancer xenograft model.

Administration Strategy ^a^	Groups	Doses (mg/kg)	Tumor Progression ^b^	
			Size Increase (x-Fold Day-0)	Significance ^c^ (*p* Values)
**Single Agents**	Vehicle	0	6.06	na
	Paclitaxel	10	4.21	ns
	Carboplatin	40	4.21	ns
	Docetaxel	10	4.13	ns
	TH1902	23	2.28	0.027
**Combinations**	Carboplatin + Paclitaxel	40 + 10	4.27	ns
	Carboplatin + Docetaxel	40 + 10	3.17	0.031
	Carboplatin + TH1902	40 + 23	1.33	0.002

^a^ Single agents were administered bi-weekly, whereas combinations were administered once at start of study. ^b^ Tumor size progression at vehicle group endpoints compared to start of treatments (Day 0). Ratios > 1 indicate tumor progression ^c^ Significance between tumor progression of individual group vs. vehicle group endpoint using one-way ANOVA with Dunnett’s multiple comparisons test, na: not applicable, ns: not significant.

## Data Availability

The data generated and/or analyzed during the current study are available from the corresponding author on reasonable request.

## Supplementary Materials

The following supporting information can be downloaded at: https://www.mdpi.com/article/10.3390/cancers14081877/s1, Figure S1. Mice weights following docetaxel and TH1902 administration in ES-2 and SKOV3 xenograft models. Mice weights monitoring following administration of docetaxel and TH1902 in ovarian cancer xenografts. Immunodeficient mice bearing subcutaneous ES-2 (A) or SKOV3 (B) tumors were intravenously injected weekly with either vehicle, docetaxel (at its MTD and quarter MTD doses; 15 and 3.75 mg/kg, respectively), or TH1902 (at a dosage which contains a quantity of bound docetaxel equal to the docetaxel doses; 35 and 8.75 mg/kg, respectively) as described in the methods section. Mice in the ES-2 study were euthanized at vehicle group endpoint (Day 18) while indicated groups in SKOV3 study were monitored for a prolonged period up to Day 46 post start of treatments. Three cycles of docetaxel at 15 mg/kg was considered as MTD. Colored dots below the abscissa indicate drug treatment events for all groups. Data are represented as percentage of initial mice weight and shown as mean ± SEM (*n* = 6 mice/group), Figure S2. Mice weights following docetaxel and TH1902 administration in AN3-CA xenograft model. Monitoring of mice weight following administration of docetaxel and TH1902 in endometrial cancer xenografts. Immunodeficient mice bearing subcutaneous AN3-CA tumors were intravenously injected weekly with either vehicle, docetaxel (at its MTD and quarter MTD doses; 15 and 3.75 mg/kg, respectively), or TH1902 (at a dosage which contains a quantity of bound docetaxel equal to the docetaxel doses; 35 and 8.75 mg/kg, respectively) as described in the methods section. Low dose docetaxel group was euthanized at vehicle group endpoint (Day 14) whereas TH1902 low dose, docetaxel and TH1902 high doses were monitored for a prolonged period (Days 21, 29 and 54 post start of treatments, respectively). Three cycles of docetaxel at 15 mg/kg was considered as MTD. Colored dots below the abscissa indicate drug treatment events for all groups, high doses of docetaxel and TH1902 were cut by half (7.5 and 17.5 mg/kg, respectively) for the third and fourth treatments because of docetaxel group weight loss (small dots). Data are represented as percentage of initial mice weight and shown as mean ± SEM (*n* = 6 mice/group), Figure S3. Mice weights following single or combined administration of TH1902, docetaxel and paclitaxel with carboplatin in an A2780 xenograft model. Monitoring of mice weights in A2780 ovarian cancer xenografts following bi-weekly administrations of (A) vehicle, docetaxel, TH1902, paclitaxel or carboplatin as single agents, or (B) administered once in combination using the same dosage as described in the methods section. Colored dots below indicate drug treatment events for all groups. Data are represented as percentage of initial mice weight and shown as mean ± SEM (*n* = 5 mice/group), Figure S4. Uncropped Western blots of Figure 3A, Figure S5. Uncropped Western blots of Figure 3B, Figure S6. Uncropped Western blots of Figure 4A.

## Abbreviations

ADC, Antibody drug conjugates; CCC, Clear cell carcinoma; CPP, Cell-penetrating peptides; EC, Endometroid carcinoma; EOC, Epithelial ovarian cancer; GC, Germ cell; HER2, Human epidermal growth factor receptor 2; MC, Mucinous carcinoma; MTD, Maximum tolerated dose; SORT1, Sortilin; TCC, Transitional cell carcinoma; TMA, Tissue microarray; HGSC, High grade serous carcinoma; LGSC, Low grade serous carcinoma; NACT, Neoadjuvant chemotherapy; NE, Non-epithelial; OCS, Ovarian carcinosarcomas; PARP, poly (ADP-ribose) polymerase; siRNA, Small interfering ribonucleic acid; VM, Vasculogenic mimicry.
