# Supplementary material for: The Peptide-Drug Conjugate TH1902: A New Sortilin Receptor-Mediated Cancer Therapeutic against Ovarian and Endometrial Cancers
Source: Cancers (Basel). 2022 Apr 8;14(8):1877. doi: 10.3390/cancers14081877 (PMC9031804; doi:10.3390/cancers14081877)
Supplement: Supplementary file 1 [file cancers-14-01877-s001.zip › cancers-1611831-supplementary.pdf]

cancers MDPI

Supplementary Materials

The Peptide–Drug Conjugate TH1902: A New Sortilin Receptor-Mediated Cancer Therapeutic against Ovarian and Endometrial Cancers

Jean-Christophe Currie [1], Michel Demeule [1], Cyndia Charfi [1], Alain Zgheib [2], Alain Larocque [1], Bogdan Alexandru Danalache [2], Amira Ouanouki [2], Richard Béliveau [2], Christian Marsolais [1] and Borhane Annabi [2,*]

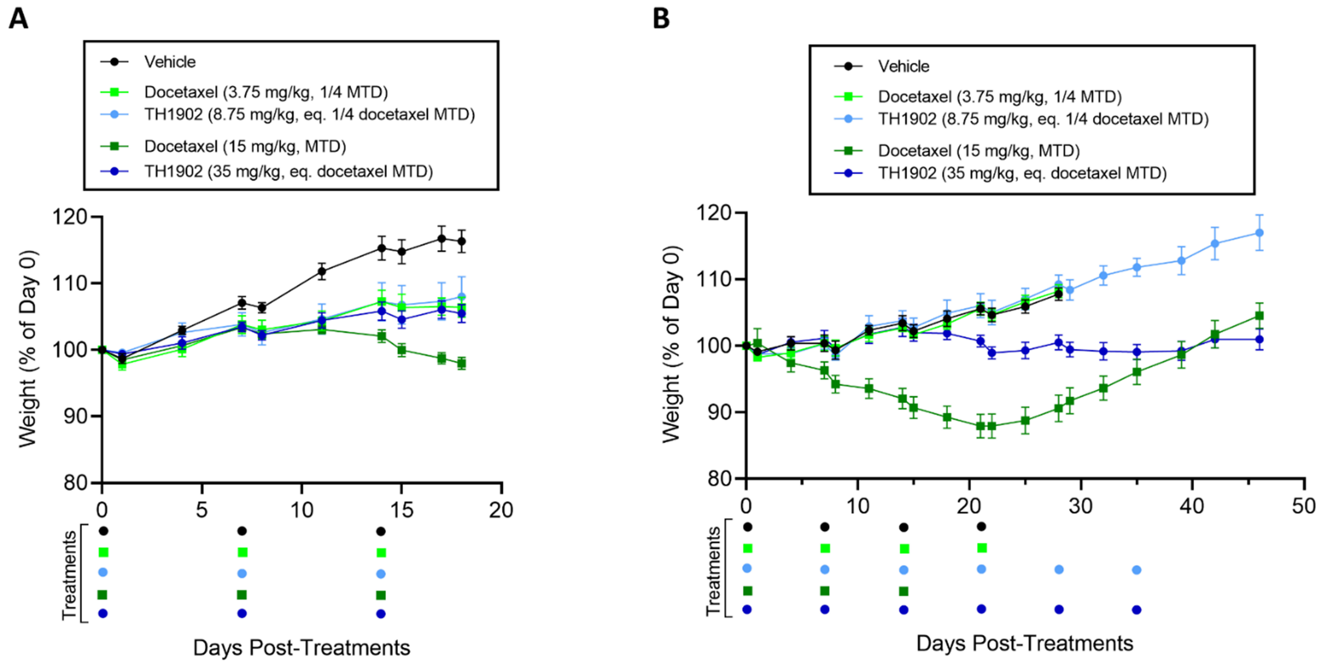

Figure S1. Mice weights following docetaxel and TH1902 administration in ES-2 and SKOV3 xenograft models. Mice weights monitoring following administration of docetaxel and TH1902 in ovarian cancer xenografts. Immunodeficient mice bearing subcutaneous ES-2 (**A**) or SKOV3 (**B**) tumors were intravenously injected weekly with either vehicle, docetaxel (at its MTD and quarter MTD doses; 15 and 3.75 mg/kg, respectively), or TH1902 (at a dosage which contains a quantity of bound docetaxel equal to the docetaxel doses; 35 and 8.75 mg/kg, respectively) as described in the methods section. Mice in the ES-2 study were euthanized at vehicle group endpoint (Day 18) while indicated groups in SKOV3 study were monitored for a prolonged period up to Day 46 post start of treatments. Three cycles of docetaxel at 15 mg/kg was considered as MTD. Colored dots below the abscissa indicate drug treatment events for all groups. Data are represented as percentage of initial mice weight and shown as mean ± SEM (n = 6 mice/group).

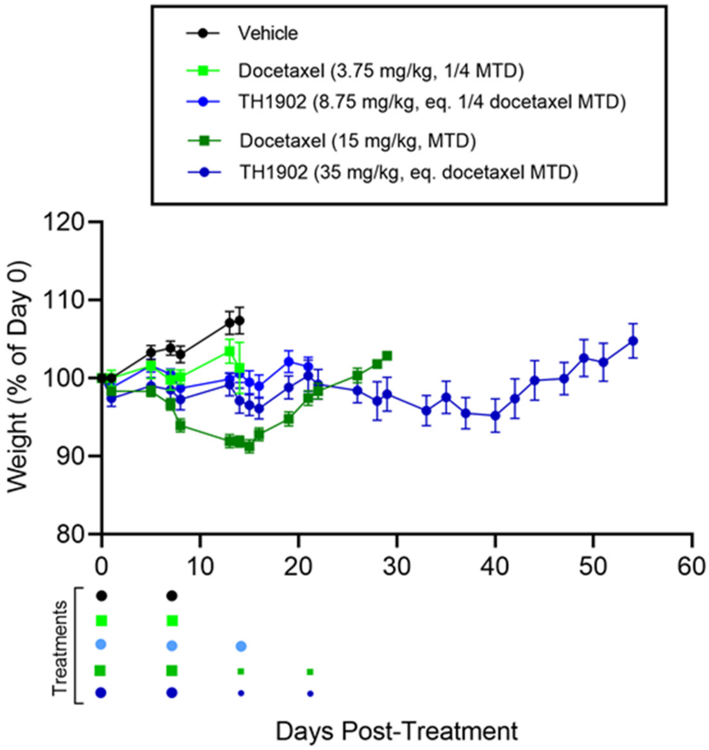

Figure S2. Mice weights following docetaxel and TH1902 administration in AN3-CA xenograft model. Monitoring of mice weight following administration of docetaxel and TH1902 in endometrial cancer xenografts. Immunodeficient mice bearing subcutaneous AN3-CA tumors were intravenously injected weekly with either vehicle, docetaxel (at its MTD and quarter MTD doses; 15 and 3.75 mg/kg, respectively), or TH1902 (at a dosage which contains a quantity of bound docetaxel equal to the docetaxel doses; 35 and 8.75 mg/kg, respectively) as described in the methods section. Low dose docetaxel group was euthanized at vehicle group endpoint (Day 14) whereas TH1902 low dose, docetaxel and TH1902 high doses were monitored for a prolonged period (Days 21, 29 and 54 post start of treatments, respectively). Three cycles of docetaxel at 15 mg/kg was considered as MTD. Colored dots below the abscissa indicate drug treatment events for all groups, high doses of docetaxel and TH1902 were cut by half (7.5 and 17.5 mg/kg, respectively) for the third and fourth treatments because of docetaxel group weight loss (small dots). Data are represented as percentage of initial mice weight and shown as mean ± SEM (n = 6 mice/group).

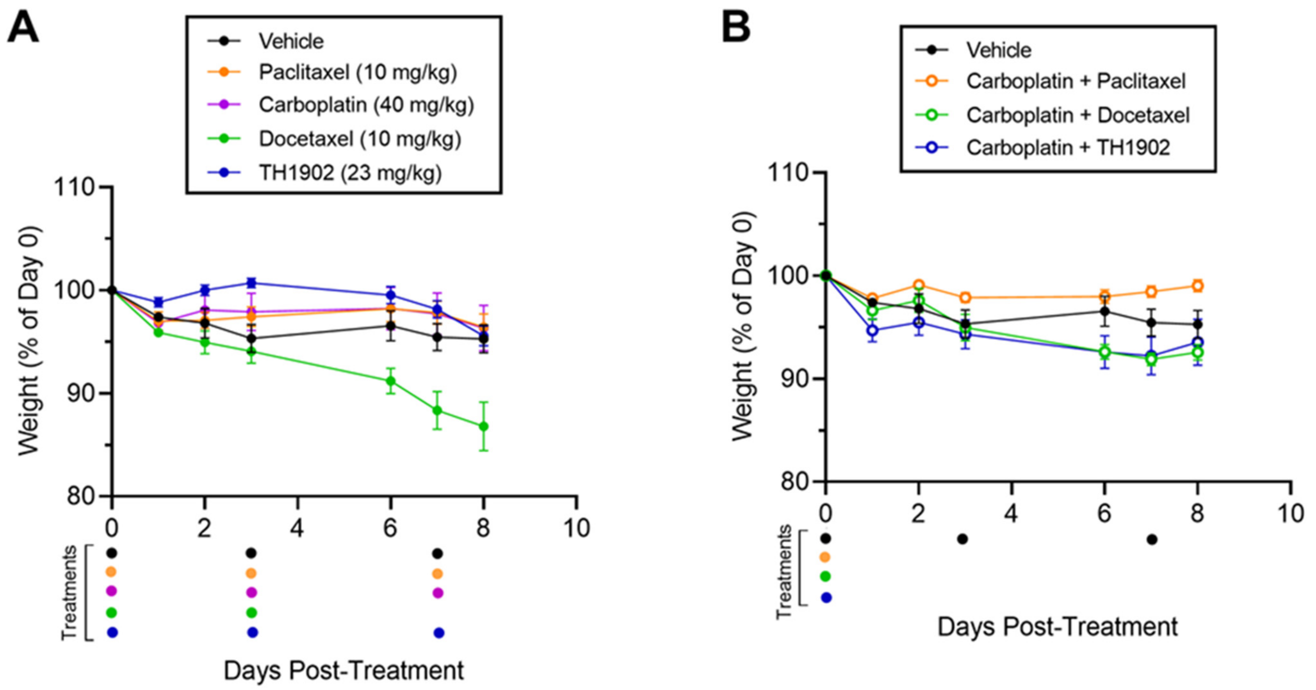

Figure S3. Mice weights following single or combined administration of TH1902, docetaxel and paclitaxel with carboplatin in an A2780 xenograft model. Monitoring of mice weights in A2780 ovarian cancer xenografts following bi-weekly administrations of (**A**) vehicle, docetaxel, TH1902, paclitaxel or carboplatin as single agents, or (**B**) administered once in combination using the same dosage as described in the methods section. Colored dots below indicate drug treatment events for all groups. Data are represented as percentage of initial mice weight and shown as mean ± SEM (n = 5 mice/group).

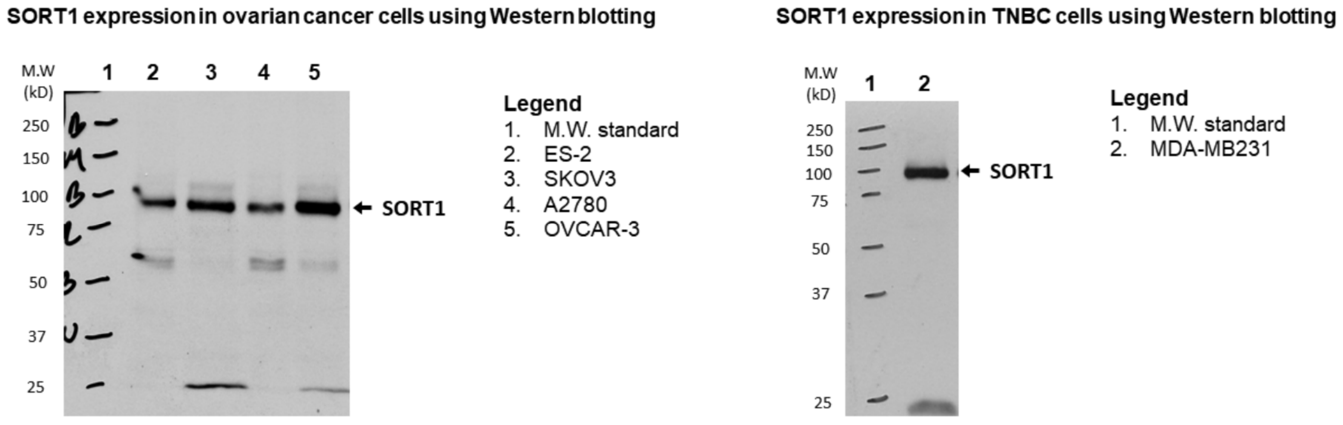

Figure S4. Uncropped Western blots of Figure 3A.

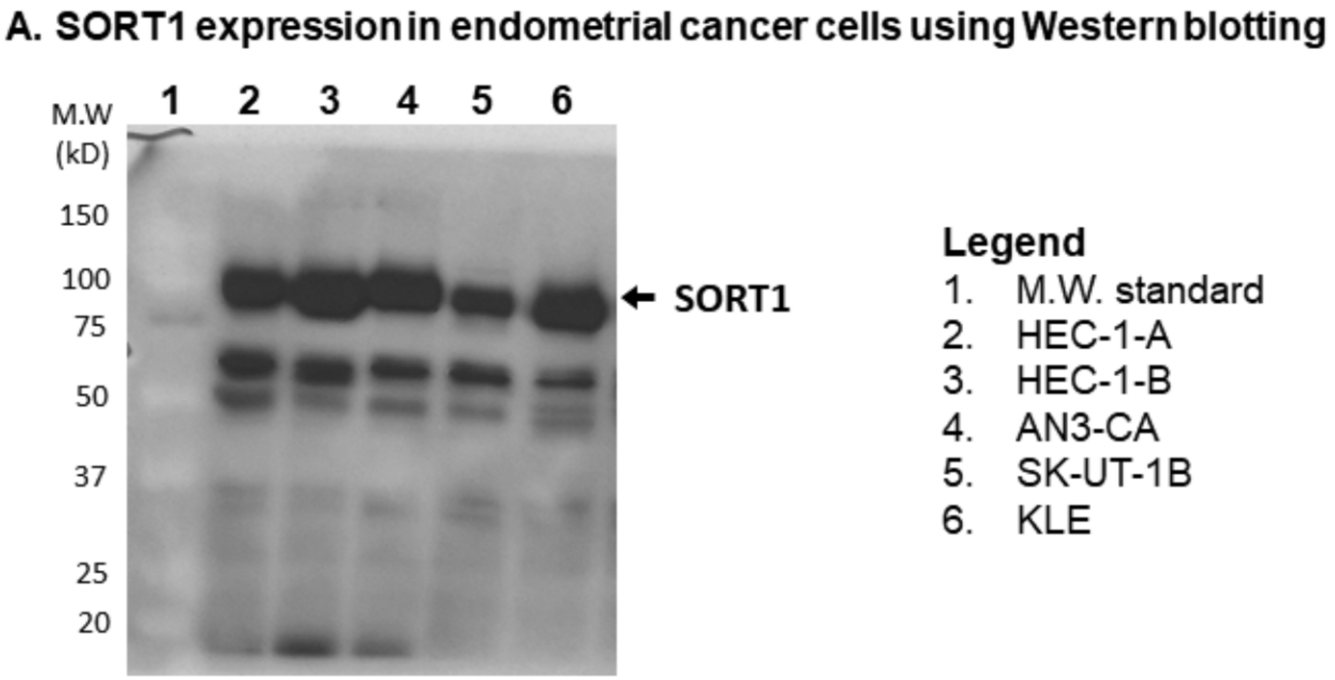

Figure S5. Uncropped Western blots of Figure 3B.

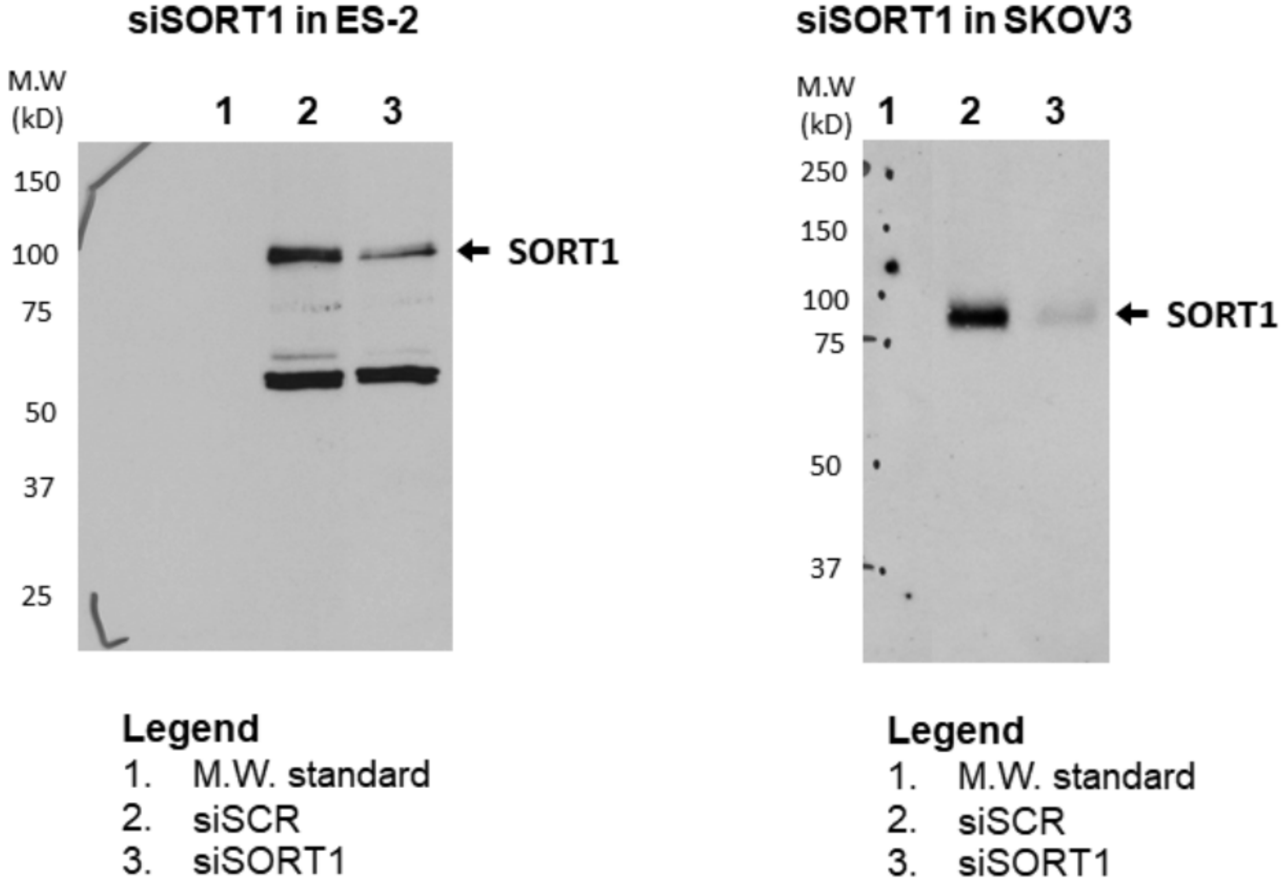

Figure S6. Uncropped Western blots of Figure 4A.
